# Long-Term Alterations of Renal Microvasculature in Rats Following Maternal PM_2.5_ Exposure: Vitamin D Effects

**DOI:** 10.3390/biomedicines13051166

**Published:** 2025-05-10

**Authors:** Eujin Park, Hyung-Eun Yim, Min-Hwa Son, Yoon-Jeong Nam, Yu-Seon Lee, Sang-Hoon Jeong, Ju-Han Lee

**Affiliations:** 1 Department of Pediatrics, Korea University Guro Hospital, 148, Gurodong-ro, Guro-gu, Seoul 08308, Republic of Korea; eujinpark@korea.ac.kr; 2 Department of Pediatrics, Korea University Ansan Hospital, 123, Jeokgeum-ro, Danwon-gu, Ansan-si 15355, Republic of Korea; isminhwa@hanmail.net; 3 Medical Science Research Center, Korea University Ansan Hospital, 123, Jeokgeum-ro, Danwon-gu, Ansan-si 15355, Republic of Korea; nyj90504@korea.ac.kr (Y.-J.N.); iknowhero@korea.ac.kr (Y.-S.L.); saibog21@korea.ac.kr (S.-H.J.); 4 Department of Pathology, Korea University Ansan Hospital, 123, Jeokgeum-ro, Danwon-gu, Ansan-si 15355, Republic of Korea; repath@korea.ac.kr

**Keywords:** ambient particulate matter, angiogenesis, fetal programming, vitamin D

## Abstract

**Background**: This study aimed to investigate the long-term effects of maternal exposure to fine particulate matter (PM_2.5_) with or without vitamin D supplementation on the renal microvasculature in adult rat offspring. **Methods**: Pregnant Sprague–Dawley rats were exposed to normal saline, PM_2.5_, and PM_2.5_ with vitamin D for one month during nephrogenesis. Male offspring kidneys were taken for analyses on postnatal day 56. **Results**: Adult offspring rats exposed to maternal PM_2.5_ exhibited lower body weights and greater glomerular and tubular injury scores compared to control rats. Semi-quantitative analysis revealed a significant reduction in glomerular and peritubular capillary endothelial cells, along with a decrease in the number of glomeruli in the PM_2.5_ group. Maternal vitamin D supplementation reduced these changes. In offspring rats exposed to maternal PM_2.5_, intrarenal expression of renin, angiotensin-converting enzyme (ACE), cytochrome P450 27B1, and vascular endothelial growth factor-A (VEGF-A) increased, while expression of the vitamin D receptor, Klotho, VEGF receptor 2, angiopoietin-1, and Tie-2 decreased. Maternal vitamin D supplementation restored VEGF receptor 2 and angiopoietin-1 activities and reduced ACE and VEGF-A protein expression in adult offspring kidneys. **Conclusions**: Early-life exposure to PM_2.5_ may lead to long-term alterations in renal microvasculature and nephron loss. Maternal vitamin D supplementation during renal development can ameliorate PM_2.5_-induced capillary rarefaction and nephron loss in the kidneys of adult offspring.

## 1. Introduction

Fine particulate matter (PM_2.5_), particles less than 2.5 μm in diameter, has emerged as a significant risk factor for the development and progression of chronic kidney disease (CKD) [1,2]. The kidneys can be vulnerable targets for PM_2.5_ exposure due to their intricate capillary networks and comprehensive endocrine functions [2,3]. Experimental studies indicate that exposure to PM_2.5_ induces oxidative stress, inflammation, endothelial dysfunction, and abnormalities in the renin-angiotensin system (RAS), which contribute to hemodynamic disturbance and ultimately lead to renal fibrosis [4,5]. Transgenerational impacts of PM_2.5_ on renal damage in offspring have been documented, establishing that in utero exposure to PM_2.5_ can predispose the offspring to kidney injury later in life [6].

Exposure of the kidney to pathogenic factors disrupts blood flow in the peritubular capillaries and interstitial region, leading to capillary loss and the development of renal fibrosis [7,8]. Vascular endothelial growth factor (VEGF)-A, a key regulator of angiogenesis, plays a critical role in renal microcirculation, with its activity being essential for the preservation and repair of capillaries in the kidney [7]. The VEGF receptor 2 (VEGFR2) mediates various VEGF-induced signal transduction pathways such as endothelial cell migration, proliferation, survival, and new blood vessel formation [9]. Therefore, the normal interaction between VEGF-A and VEGFR2 in the kidney is crucial for maintaining the integrity of the renal microvasculature [10]. Klotho, an antiaging protein, is abundantly expressed in renal tubular cells and inhibits the expression of all RAS components within the kidney [11]. It functions as a co-receptor for fibroblast growth factor-23 and is involved in the metabolism of phosphate and vitamin D [12]. Additionally, Klotho contributes to VEGF-mediated vascular functions and the preservation of endothelial integrity [13,14]. Angiopoietin (Ang)-1 and Ang-2, members of a family of endothelial growth factors, are also essential for maintaining the homeostasis of the kidney vasculature [15]. The binding of Ang-1 to the tyrosine kinase receptor Tie-2 facilitates anti-inflammatory and pro-angiogenic activities, whereas Ang-2 has opposing effects [16]. Alongside VEGF-A, Angs are implicated in renovascular growth and are crucial for endothelial differentiation, survival, and repair capacity [17]. In rats, postnatal RAS inhibition during nephrogenesis decreased renal expression of Angs, Tie-2, and VEGF-A [18], while angiotensin II stimulation increased renal VEGF-A expression both in vitro and in vivo [19,20]. A lack of vitamin D during renal development caused glomerular and peritubular capillary loss, associated with an imbalance between pro- and antiangiogenic factors and elevated intrarenal renin levels in adult offspring rats [21]. Vitamin D treatment improved renal microvascular rarefaction by regulating the Angs/Tie-2, VEGF/VEGFR2, and RAS pathways, which were disrupted in an animal model of CKD [22]. The authors propose that vitamin D could be a promising target for alleviating renal microvascular impairment at the onset of CKD [22].

Various renal programming models demonstrate a biphasic intrarenal RAS response, with downregulation of the classical RAS during the neonatal period, followed by inappropriate activation in adulthood [23,24]. Early-life insults can suppress the RAS during development, hinder its normalization, and lead to its abnormal activation later in life, contributing to renal injuries and nephron loss [23,24]. We previously demonstrated that maternal exposure to PM_2.5_ during nephrogenesis results in kidney injury in both dams and pups, likely due to disturbances in vitamin D signaling and the RAS [25]. This study aimed to investigate whether maternal exposure to PM_2.5_ during renal development adversely affects renal microvascular homeostasis in the kidneys of adult offspring over the long term. Furthermore, we assessed the effects of maternal vitamin D supplementation on PM_2.5_-induced renal vascular alterations in adult rat offspring.

## 2. Materials and Methods

### 2.1. PM_2.5_ and Animal Preparation

Airborne PM_2.5_ samples were collected using a high-volume air sampler (HV-1700RW, Sibata, Tokyo, Japan) on the rooftop of Korea University Ansan Hospital, in Ansan-si, Gyeonggi-province, South Korea [25,26]. Two filters collected on December 22, 2019, above the World Health Organization guidelines for PM_2.5_ (≥15 μg/m^3^ per day) were chosen. Real-time air quality data (https://www.airkorea.or.kr/eng (accessed on 22 March 2022)). were used to verify PM_2.5_ levels. After drying the filters in an automatic dryer (Sanpla Dry Keeper, Sanplec Corp., Osaka, Japan), they were cut into 2 × 2 cm^2^ pieces, immersed in 100 mL phosphate-buffered saline, and sonicated three times for 30 min. The suspension was then shaken for 10 min and filtered through a 0.2 μm syringe filter to obtain fine particles. The following equation, based on the average daily dose calculation of the U.S. Environmental Protection Agency, was used to estimate PM_2.5_ exposure [27]:

Average daily dose (mg/kg·day) = (Cair × InhR × ET × EF × ED) / (BW × AT).

Cair, InhR, ET, EF, ED, BW, and AT represent the contaminant concentration in air (mg/m^3^), inhalation rate (m^3^/day), exposure time (hours/day), exposure frequency (days/year), exposure duration (years), body weight (kg), and average time (days), respectively. To induce maternal exposure, each dam received 70 μL of PM_2.5_ suspension five times per week for four weeks (total 1.47 mL, 70 μL × 21 doses). For a 60 kg human, the average respiratory volume over 23 h is 9660 L/day (500 mL tidal volume × 14 breaths/min × 60 min × 23 h) [27]. The air sampler operated at 1000 L/min for 23 h, collecting 1,380,000 L/day. Therefore, two filters corresponded to 286 days of human respiration (1,380,000 L/day × 2 ÷ 9660 L/day). Particles from the two filters were suspended in 50 mL of normal saline (NS), yielding 0.175 mL as the estimated daily exposure (50 mL ÷ 286 days). Thus, the total volume administered to each dam (1.47 mL) was equivalent to approximately 8.4 days of human PM_2.5_ exposure (1.47 mL ÷ 0.175 mL/day) [25].

**Figure 1:**
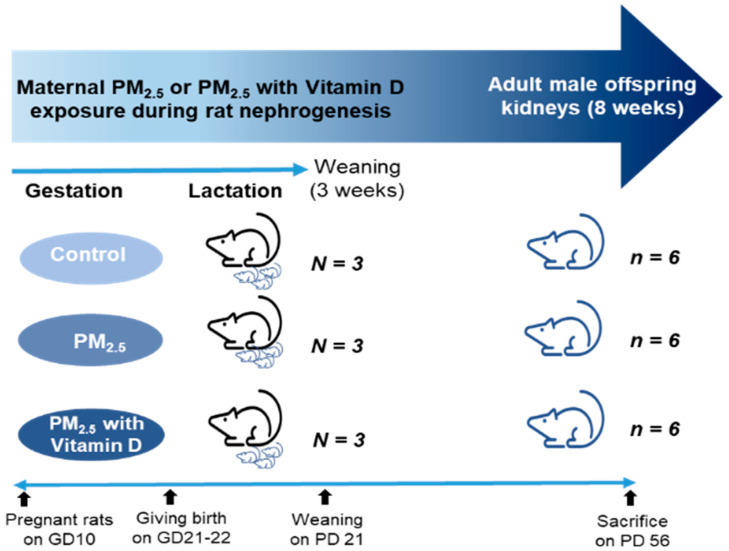
Experimental design. GD, gestational day; PD, postnatal day.

For the animal studies, nine pregnant Sprague–Dawley rats on gestation day 10 (Raonbio, Yong-in, South Korea) were kept under standard conditions. All pregnant rats were allowed natural birth, with pups being born on days 21–22 of gestation. The pregnant rats were randomly divided into three groups: control, PM_2.5_, and PM_2.5_ with vitamin D (PV) groups (*N* = 3 in each group). They received either NS (70 μL, 5 times/week), PM_2.5_ (70 μL dissolved in saline, 5 times/week), or PM_2.5_ (70 μL dissolved in saline, 5 times/week) with vitamin D (cholecalciferol, 1000 IU/kg, 3 times/week; FND Net Co., Seoul, South Korea) via an orogastric tube from gestation day 11 to lactation day 21. The pups were weaned on postnatal day 21 and remained without intervention until postnatal day 56. The body weights of male offspring rats were measured every 3 to 7 days. Male adult offspring (*n* = 6 per each dam group) were euthanized on postnatal day 56. Given that many studies demonstrate male offspring are particularly susceptible to fetal insults during critical developmental windows [28], only male offspring rats were utilized in our experiments. The adult male offspring rats were anesthetized with 2% isoflurane, and blood was drawn to assess levels of calcium (Ca^2+^), 25-hydroxyvitamin D [25(OH)D], and cystatin C via cardiac puncture. Their kidneys were then extracted for Western blot analysis (right kidneys) and histological and immunohistochemical evaluations (left kidneys). The experimental design is illustrated as follows (Figure 1).

### 2.2. Histological Examination

Kidney tissues were fixed in 10% formalin, embedded in paraffin, and then sectioned to a thickness of 4 μm. Hematoxylin and eosin (H&E) and periodic acid–Schiff (PAS) staining were utilized to ascertain renal structural alterations. Glomerular damage, which includes mesangial expansion, glomerular tuft destruction, and glomerulosclerosis, was scored (0 to 4) following the methodology described by Raij et al. (0 indicates no lesion; 1, <25% of glomerulus damaged; 2, 25–50%; 3, 50–75%; and 4, >75%) from H&E stained slide images [29]. Tubulointerstitial injury, encompassing tubular dilation, atrophy, cast formation, tubular cell flattening, and interstitial cell infiltration, was similarly scored from PAS-stained slide images using the same scale. The extent of glomerular damage and tubulointerstitial injury was assessed across ten randomly selected fields per kidney in five rats at 200× magnification using a double-blind method. To determine glomerular damage accurately, at least 20 glomeruli per section were examined. All stained slides were digitized using Pannoramic Scan II (3DHISTECH, Sysmex, Budapest, Hungary) and analyzed on digital images (CaseViewer, 3DHISTECH, version 2.2.0).

### 2.3. Western Blotting

Kidney tissues were homogenized using the T-PER™ Tissue Protein Extraction Reagent (Thermo Scientific, Rockford, IL, USA) and the Xpert Protease Inhibitor Cocktail Solution (100×) (GenDEPOT, Katy, TX, USA). Protein concentrations were determined via the bicinchoninic acid assay. Equal amounts of proteins (20 μg) were separated on 8–15% SDS-PAGE and transferred to polyvinylidene fluoride membranes. The membranes were incubated overnight at 4 °C with primary antibodies against vitamin D receptor (VDR) (Abcam, Cambridge, MA, USA), Klotho (Invitrogen, Carlsbad, CA, USA), cytochrome P450 mixed-function oxidase (CYP)27B1 (Abcam), CYP24A1 (Invitrogen), renin (Santa Cruz biotechnology, Santa Cruz, CA, USA), angiotensin-converting enzyme (ACE) (Invitrogen), angiotensin II receptor type 1 (AT1) (Invitrogen), VEGF-A (Santa Cruz Biotechnology), VEGFR2 (Santa Cruz Biotechnology), hypoxia-inducible factor-1 alpha (HIF-1α) (Santa Cruz Biotechnology), Ang-1 (Santa Cruz Biotechnology), Tie-2 (Santa Cruz Biotechnology), Ang-2 (Santa Cruz Biotechnology), and thrombospondin (TSP)-1 (Santa Cruz Biotechnology). Subsequently, the blots were incubated with secondary antibodies (peroxidase-conjugated anti-rabbit or anti-mouse IgG; Cell Signaling Technology, Danvers, MA, USA) at room temperature for 2 h. β-actin (Santa Cruz Biotechnology) was used as a housekeeping gene. The ChemiDoc Touch Imaging System (Bio-Rad Laboratories, Hercules, CA, USA) was used to image and analyze the detected Western blots.

### 2.4. Immunohistochemistry (IHC)

For IHC, kidney sections were deparaffinized and incubated with the following antibodies: VDR (Santa Cruz Biotechnology), Klotho (Invitrogen), CYP27B1 (Invitrogen), renin (Santa Cruz Biotechnology), ACE (Invitrogen), VEGF-A (Novus, Centennial, CO, USA), VEGFR2 (Cell Signaling), Ang-1 (Invitrogen), and Tie-2 (Abcam). Antigen retrieval was achieved using Tris-EDTA pH 8.0 buffer, and all slides were stained with a Dako AutoStainer™ (Dako, Carpinteria, CA, USA). The signals were visualized using the Novolink Polymer Detection System (RE7150-K; Leica Biosystems, Newcastle Upon Tyne, UK). IHC against aminopeptidase P antibody (JG12, an endothelial cell marker) (Invitrogen) was also performed to evaluate capillary density. Glomerular and peritubular capillary density were determined, respectively, using a point detection method in ten random 10 × 10 grids of cortex per section from each of five rats at 200× magnification in a blinded manner. For semi-quantification of glomerular capillary density, the proportion was calculated by dividing the number of glomeruli showing positive JG-12 grid points by the total number of glomeruli within the grid. At least 50 glomeruli were examined for each rat. Pannoramic Scan II (3DHISTECH, Sysmex, Budapest, Hungary) and CaseViewer (3DHISTECH, version 2.2.0) software programs were also employed for digitalizing, viewing, and analyzing all stained slides.

### 2.5. Biochemical Analysis

Renal function in adult offspring rats was assessed by measuring the levels of serum cystatin C. Vitamin D status and calcium levels were evaluated through the analysis of serum 25(OH)D concentrations and Ca^2+^ concentrations, respectively. Collected blood on postnatal day 56 was centrifuged at 3000 rpm for 15 min, and serum was stored at −80 °C until analysis. Concentrations of Ca^2+^, 25(OH)D, and cystatin C in the blood were determined using commercial enzyme-linked immunosorbent assay (ELISA) kits: a calcium assay kit (Abcam), a 25(OH)D ELISA kit (MyBioSource, San Diego, CA, USA), and a rat cystatin C quantikine ELISA kit (R&D Systems, Minneapolis, MN, USA).

### 2.6. Glomerular Counting

At 8 weeks of age, glomerular numbers were quantified using 4 μm thick tissue sections obtained from six kidneys per group. In each section, the number of glomeruli was counted in 10 randomly selected, non-overlapping fields within a standardized 10 × 10 grid under 100×magnification across four sections stained with H&E and PAS. Counts were performed in a double-blinded manner by two independent investigators. In total, 240 fields were analyzed per group (6 kidneys × 4 sections × 10 fields). The mean ± standard deviation was calculated for each group, and statistical significance among the groups was evaluated.

### 2.7. Statistical Analyses

All experiments were conducted at least three times, and results were presented as means ± standard error of the mean or standard deviation. Statistical analyses were performed using GraphPad Prism v.7.0 (Graphpad Software Inc., San Diego, CA, USA). Data were analyzed using one-way analysis of variance with multiple comparisons test. *p* < 0.05 was considered statistically significant.

## 3. Results

### 3.1. Body Weight Changes and Laboratory Data

**Figure 2:**
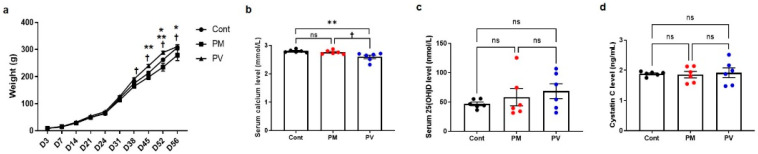
Offspring body weights and biochemical analysis: (**a**) Changes in body weight up to postnatal day 56. (**b**) Serum calcium levels. (**c**) Serum 25(OH)D concentrations. (**d**) Serum cystatin C levels on postnatal day 56 in offspring rats. * *p* < 0.05, Cont vs. PM; ** *p* < 0.05, Cont vs. PV; † *p* < 0.05, PM vs. PV (*n* = 6/group). Cont, control group; PM, PM group; PV, PV group; ns, not significant.

Male offspring rats exhibited similar body weights across three groups from birth until postnatal day 31. However, adult rats subjected to maternal PM_2.5_ exhibited lower body weights compared to those from the control group and the PV group on postnatal days 52 and 56, respectively (all *p* < 0.05, PM_2.5_ vs. control or PV). Offspring of the maternal PV group exhibited higher body weights compared to those from the PM_2.5_ group from postnatal day 38 to 56 (all *p* < 0.05, PV vs. PM_2.5_) (Figure 2a). Serum calcium levels were similar between offspring of the control group and those of the PM_2.5_ group, but offspring of the PV group displayed lower calcium levels than the other two groups (*p* < 0.05, PV vs. control or PM_2.5_) (Figure 2b). Levels of serum 25(OH)D and cystatin C did not differ among the groups (Figure 2c,d).

### 3.2. Renal Histological Alterations

**Figure 3:**
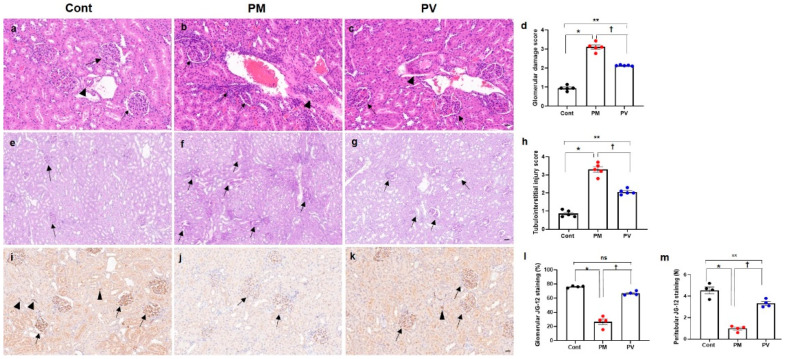
Renal histological changes and capillary density in adult offspring kidneys: (**a**–**c**) H&E staining. (**d**) Glomerular damage scores. (**e**–**g**) PAS staining. (**h**) Tubulointerstitial injury scores. (**i**–**k**) JG-12 immunostaining images. (**l**) Quantification of glomerular JG-12 immunostaining. (**m**) Quantification of peritubular JG-12 immunostaining. Adult offspring exposed to maternal PM_2.5_ showed glomerular, tubulointerstitial, and vascular changes ((**b**,**f**) arrows, glomerular retraction and tubulointerstitial infiltrates; arrowhead, abnormal vascular morphology with wall thickening) compared to control kidneys ((**a**,**e**) arrows, glomeruli; arrowhead, vessel). Maternal vitamin D supplementation attenuated these renal structural alterations in offspring rats ((**c**,**g**) arrows, abnormal glomeruli and tubules; arrowhead, mildly thickened vessel). While glomerular (arrows) and peritubular (arrowheads) capillary endothelial cells were well preserved in control rats (**i**), capillary rarefaction (arrows) was pronounced in the maternal PM_2.5_-exposed offspring kidneys (**j**). Maternal vitamin D supplementation reduced the loss of glomerular (arrows) and peritubular (arrowheads) capillaries (**k**) in adult offspring kidneys. * *p* < 0.05, Cont vs. PM; ** *p* < 0.05, Cont vs. PV; † *p* < 0.05, PM vs. PV ((**a**–**c**) and (**i**–**k**) 400×, bar = 20 µm; (e-g) 200×, bar = 50 µm) (*n* = 4–5/group). Cont, control group; PM, PM group; PV, PV group; ns, not significant.

For the evaluation of renal structural changes, we conducted H&E (Figure 3a–c) and PAS staining (Figure 3e–g). Exposure to maternal PM_2.5_ during pregnancy and lactation induced glomerular, tubulointerstitial, and vascular alterations in the kidneys of adult offspring rats. Histological analysis revealed increased scores of glomerular damage and tubulointerstitial injury, including glomerular capillary retraction, mesangial expansion, tubular dilatation, cast formation, and interstitial inflammatory infiltrates, as well as vascular abnormalities in the groups exposed to maternal PM_2.5_ (*p* < 0.05, PM_2.5_ vs. control), which were alleviated by maternal vitamin D supplementation (*p* < 0.05, PM_2.5_ vs. PV). Offspring rats from the maternal PV group still displayed higher renal structural injury scores than those from the control group (*p* < 0.05, PV vs. control) (Figure 3d,h).

### 3.3. Intrarenal Capillary JG12 Expression

We investigated the effects of maternal PM_2.5_ exposure, with or without vitamin D, on capillary rarefaction in the kidneys of adult offspring (Figure 3i–m). Exposure to PM_2.5_ reduced endothelial expression of glomerular and peritubular JG-12 (both *p* < 0.05, PM_2.5_ vs. control), while maternal vitamin D supplementation reversed this effect in offspring rat kidneys (both *p* < 0.05, PV vs. PM_2.5_). Although glomerular capillary JG-12 staining was similar between the adult offspring rats from the PV and control groups (Figure 3l), peritubular capillary loss was greater in those from the maternal PV group compared to the controls (Figure 3m).

### 3.4. Intrarenal VDR, Klotho, CYP27B1, and CYP241A Expression

**Figure 4:**
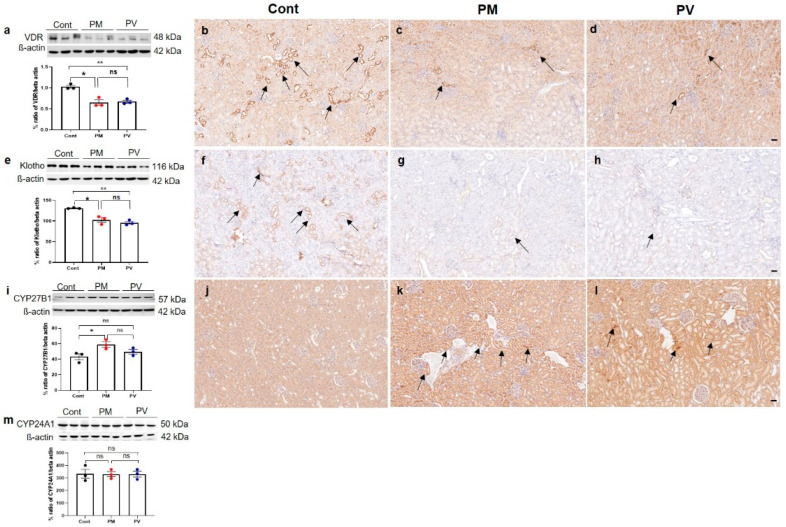
Intrarenal vitamin D signaling in adult offspring kidneys: (**a**–**d**) VDR; (**e**–**h**) Klotho; (**i**–**l**) CYP27B; (**m**) CYP24A1. Western blot analysis revealed decreases in VDR (**a**) and Klotho (**e**) activities in kidneys of offspring exposed to maternal PM_2.5_ compared to controls; these were not restored by maternal vitamin D treatment. Control rats showed prominent expressions of VDR ((**b**), arrows) and Klotho ((**f**), arrows) in tubular epithelial cells, whereas rats in the PM and PV groups exhibited reduced expressions ((**c**,**g**), PM group, arrows; (**d**,**h**), PV group). Compared to the control rats (**j**), CYP27B1 expression was more pronounced in the tubular cells of the PM ((**k**), arrows) and PV groups ((**l**), arrows). No differences in CYP24A1 (**m**) activities were observed among the groups (* *p* < 0.05, Cont vs. PM; ** *p* < 0.05, Cont vs. PV) (200×, bar = 50 µm) (*n* = 3–6/group). Cont, control group; PM, PM group; PV, PV group; ns, not significant.

We then assessed changes in the vitamin D signaling pathway in adult offspring rat kidneys (Figure 4). Maternal exposure to PM_2.5_ suppressed the intrarenal protein expression of VDR (Figure 4a–d) and klotho (Figure 4e–h) (both *p* < 0.05, PM_2.5_ vs. control), and these reductions were not reversed by vitamin D treatment. Immunohistochemically, both VDR and klotho expressions predominantly in the tubular epithelial cells were observed in offspring kidneys from the control group but were weakly detected in those from the PM_2.5_ or PV groups. Conversely, protein expression of CYP27B1 increased in the maternal PM_2.5_ exposure group (*p* < 0.05, PM_2.5_ vs. control) and was not reversed by maternal vitamin D supplementation (Figure 4i–l). The intrarenal expression of CYP24A1 showed no significant differences among the groups (Figure 4m).

### 3.5. Intrarenal Renin, ACE, and AT1 Expression

**Figure 5:**
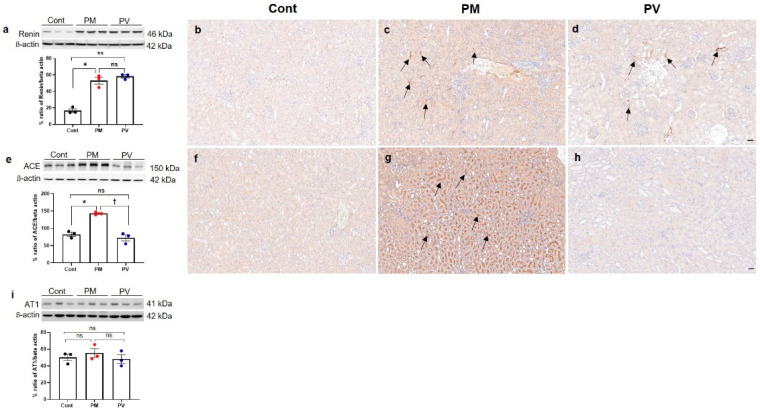
Intrarenal RAS signaling in adult offspring kidneys: (**a**–**d**) renin. (**e**–**h**) ACE. (**i**) AT1. Western blot analysis showed increased renin (**a**) and ACE (**e**) activities in the kidneys of offspring rats exposed to maternal PM_2.5_ compared to controls. Maternal vitamin D supplementation significantly reduced intrarenal ACE activity (**e**). Immunohistochemically, renin expression was observed in the interstitium and juxtaglomerular cells of offspring kidneys from the PM ((**c**), arrows) and PV groups ((**d**), arrows), whereas it was rarely detected in control offspring kidneys (**b**). ACE was also expressed extensively throughout the brush border of tubular cells in kidneys of offspring exposed to maternal PM_2.5_ ((**g**), arrows). Maternal vitamin D supplementation markedly reduced intrarenal ACE expression in the adult offspring rats (**h**). No differences were observed in intrarenal AT1 activity among the groups (**i**) (* *p* < 0.05, Cont vs. PM; ** *p* < 0.05, Cont vs. PV; † *p* < 0.05, PM vs. PV) (200×, bar = 50 µm) (*n* = 3–6/group). Cont, control group; PM, PM group; PV, PV group; ns, not significant.

To investigate the alterations in intrarenal RAS signaling in adult offspring rats, we assessed the levels of intrarenal renin, ACE, and AT1 (Figure 5). Maternal exposure to PM_2.5_ significantly elevated the protein expression of renin and ACE in the kidneys of adult offspring (both *p* < 0.05, PM_2.5_ vs. control). Although maternal vitamin D intake did not reverse the renin expression induced by PM_2.5_ exposure (*p* < 0.05, PV vs. control) (Figure 5a–d), it significantly decreased intrarenal ACE activity in adult offspring rat kidneys (*p* < 0.05, PV vs. PM_2.5_) (Figure 5e–h). Renin expression was prominently observed in the interstitium of offspring kidneys from both PM_2.5_ and PV groups (Figure 5c,d). Furthermore, ACE expression was clearly present on most luminal surfaces of tubules in the maternal PM_2.5_ exposure group, which was diminished by maternal vitamin D supplementation (Figure 5f–h). However, AT1 expression remained unchanged among the groups (Figure 5i).

### 3.6. Intrarenal VEGF-A, VEGFR2, and HIF-1α Expression

**Figure 6:**
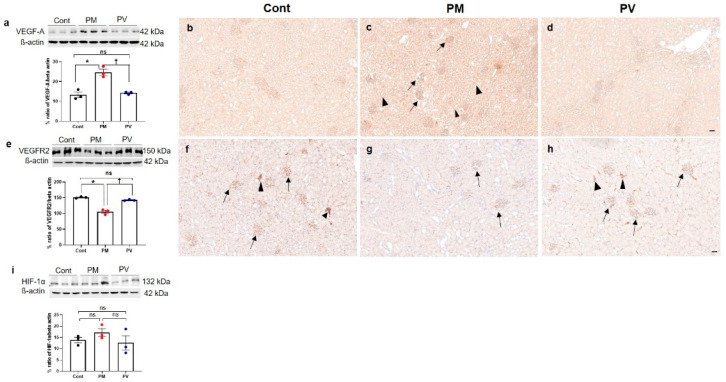
Intrarenal VEGF-A, VEGFR2, and HIF-1α expressions in adult offspring kidneys: (**a**–**d**) VEGF-A. (**e**–**h**) VEGFR2. (**i**) HIF-1α. Western blot analysis revealed an increase in VEGF-A (**a**) and a decrease in VEGFR2 (**e**) activity in maternal PM_2.5_-exposed offspring’s kidneys compared to the controls, which was reversed by maternal vitamin D treatment. Intrarenal VEGF-A expression was strongly detected throughout almost all tubular segments and glomeruli in the PM_2.5_ group ((**c**), arrows, glomeruli; arrowheads, tubules), compared to the controls (**b**) and the PV group (**d**). While VEGFR2 expression was prominently observed in glomeruli and peritubular capillaries in control kidneys ((**f**), arrows, glomeruli; arrowheads, peritubular capillaries and interstitium), it was weakly detected in offspring kidneys from maternal PM_2.5_ exposure ((**g**), arrows, glomeruli). Maternal vitamin D intake restored VEGFR2 expression in glomeruli and interstitium ((**h**), arrows, glomeruli; arrowheads, peritubular capillaries and interstitium). Intrarenal activity of HIF-1α was not different among the groups (**i**) (* *p* < 0.05, Cont vs. PM; † *p* < 0.05, PM vs. PV) (200×, bar = 50 µm) (*n* = 3–6/group). Cont, control group; PM, PM group; PV, PV group; ns, not significant.

Next, we investigated the impact of maternal PM_2.5_ exposure with or without vitamin D supplementation on renal angiogenesis in adult offspring rats. We first assessed the protein expressions of HIF-1α, VEGF-A, and its receptor VEGFR2 (Figure 6). Exposure to PM_2.5_ during nephrogenesis led to increased VEGF-A activity (Figure 6a–d) in offspring kidneys while diminishing VEGFR2 expression (both *p* < 0.05, PM_2.5_ vs. control). Maternal vitamin D supplementation effectively reversed these changes in the kidneys of adult offspring (both *p* < 0.05, PV vs. PM_2.5_) (Figure 6e–h). Immunohistochemically, VEGF-A was prominently expressed in most glomeruli and tubules in the offspring kidneys of the maternal PM_2.5_ group compared to the control group. Conversely, intrarenal VEGFR2 expression was reduced in the offspring rats from the PM_2.5_ group. It was abundantly detected in most peritubular capillaries and glomeruli in the offspring kidneys from the control group. Maternal vitamin D treatment diminished VEGF-A expression and restored VEGFR2 activity. However, maternal PM_2.5_ exposure, with or without vitamin D, did not influence intrarenal HIF-1α expression in adult offspring rats (Figure 6i).

### 3.7. Intrarenal Ang-1, Ang-2, Tie-2 and TSP-1 Expression

**Figure 7:**
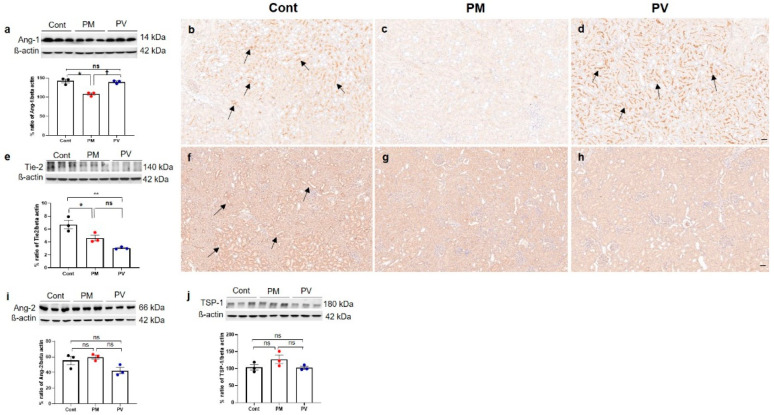
Intrarenal Angs, Tie-2, and TSP-1 expressions in adult offspring kidneys: (**a**–**d**) Ang-1. (**e**–**h**) Tie-2. (**i**) Ang-2. (**j**) TSP-1. Western blot analysis showed reductions in Ang-1 (**a**) and Tie-2 (**e**) activities in kidneys of offspring exposed to maternal PM_2.5_ compared to controls. Maternal vitamin D intake restored Ang-1 expression in the offspring’s kidneys. Immunohistochemically, Ang-1 (**b**) and Tie-2 (**f**) expressions were clearly observed in tubular cells in the controls (arrows). However, they were rarely detected in offspring kidneys exposed to maternal PM_2.5_ (**c**,**g**). Intrarenal activity of Ang-1 in offspring kidneys increased with maternal vitamin D supplementation ((**d**), arrows, tubular cells). There were no differences in intrarenal activities of Ang-2 (**i**) and TSP-1 (**j**) among the groups (* *p* < 0.05, Cont vs. PM; ** *p* < 0.05, Cont vs. PV; † *p* < 0.05, PM vs. PV) (200×, bar = 50 µm) (*n* = 3–6/group). Cont, control group; PM, PM group; PV, PV group; ns, not significant.

We subsequently explored the Ang-Tie signaling pathway, which also regulates angiogenesis and vascular homeostasis [15] (Figure 7). In the maternal PM_2.5_ exposure group, intrarenal expressions of Ang-1 (Figure 7a–d) and Tie-2 (Figure 7e–h) were diminished compared to the controls (both *p* < 0.05, PM_2.5_ vs. control). While Ang-1 expression was increased by maternal vitamin D supplementation (*p* < 0.05, PV vs. PM_2.5_), Tie-2 activity was not restored in the PV group (*p* < 0.05, PV vs. control). On IHC, Ang-1 and Tie-2 were primarily observed in the tubular epithelial cells in the control group (Figure 7b,f). Significant reductions in tubular Ang-1 and Tie-2 expression were noted in the PM_2.5_ group (Figure 7c,g), and maternal vitamin D supplementation only reversed Ang-1 expression (Figure 7d). Ang-2 and TSP-1 expressions remained unchanged across the groups (Figure 7i,j).

### 3.8. Glmerular Number

**Figure 8:**
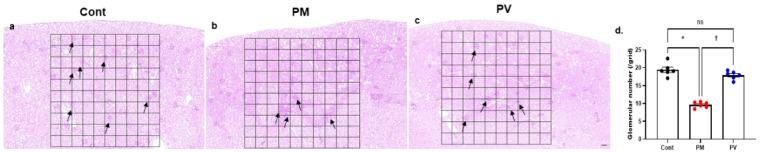
Representative photomicrographs and glomerular counts from kidney sections: (**a**–**c**) PAS staining. (**a**) control group, (**b**) PM_2.5_-exposed group, (**c**) PV group, and (**d**) glomerular number per grid. Glomeruli were counted only if they were fully enclosed within a 10 × 10 grid in the cortical region. Offspring exposed to PM_2.5_ exhibited a significantly reduced number of glomeruli per grid, which was ameliorated by maternal vitamin D supplementation (*n* = 6 kidneys × 4 sections × 10 fields for each group) (* *p* < 0.05, Cont vs. PM; † *p* < 0.05, PM vs. PV) (100×, bar = 100 µm). Cont, control group; PM, PM group; PV, PV group; ns, not significant.

Finally, the number of nephrons in each group was estimated by counting glomeruli within a standardized grid across kidney sections stained with H&E and PAS (Figure 8). Maternal exposure to PM_2.5_ during the nephrogenic period resulted in a decreased number of glomeruli in 8-week-old offspring rats (Figure 8b,d). Notably, maternal vitamin D supplementation ameliorated this effect, leading to an increased glomerular count in the adult offspring (Figure 8c,d).

## 4. Discussion

This study demonstrated that maternal exposure to PM_2.5_ during renal development induced glomerular and tubular injuries, capillary rarefaction, nephron loss, and downregulation of angiogenic factors in the kidneys of adult offspring rats. These adverse effects were associated with disruptions in intrarenal RAS and vitamin D signaling pathways. Maternal vitamin D supplementation during PM_2.5_ exposure lessened these alterations in adult offspring rat kidneys. Early life exposure to ambient PM_2.5_ may predispose to long-term kidney injury, manifesting as renal microvascular disturbances and nephron deficits. Maternal vitamin D intake during fetal kidney development could, in part, protect against renal damage and diminish the antiangiogenic environment caused by in utero PM_2.5_ exposure in adult offspring.

Maternal exposure to air pollution during pregnancy can adversely affect fetal growth and the long-term health of offspring [30,31]. In utero PM exposure increases the risk of low birth weight and preterm birth and is reported to promote adult cardiovascular disease [30,31]. This study showed that exposure to fine particulate air pollution during nephrogenesis in rats induced lower body weight in adult offspring. While birth weights were similar across groups, adult offspring in the maternal PM_2.5_ exposure group gained less weight than the controls. This group also exhibited renal structural changes, including capillary loss and a decreased glomerular count in adulthood, although changes in kidney function were not clearly evident. While the effects of maternal PM_2.5_ exposure on nephron endowment remain underexplored, our findings demonstrate a reduction in glomerular number in adult offspring. Consistent with this observation, in utero exposure to maternal smoking has been shown to impair renal development, resulting in sustained nephron deficits, reduced fetal kidney size and altered renal microarchitecture [32,33]. Notably, the weight of offspring from dams with vitamin D supplementation during PM_2.5_ exposure was consistently higher than that from the maternal PM_2.5_ exposure group, with a significant difference from 5 weeks of age. Maternal vitamin D intake also attenuated the glomerular and tubulointerstitial damage, microvascular rarefaction and glomerular loss caused by PM_2.5_ exposure in adult offspring rats. Given the potential benefits of vitamin D on offspring health outcomes during pregnancy [34], maternal vitamin D supplementation may help alleviate poor weight gain and renal structural disturbances associated with perinatal PM_2.5_ exposure.

The RAS is highly expressed in the developing kidney and plays a decisive role in renal programming [23]. It is well established that the use of RAS blockers during nephrogenesis results in nephron loss, hypertension, and CKD later in life [35,36,37]. Intrarenal RAS is regulated by complex networks that comprise positive regulators, including the Wnt/ß-catenin signaling pathway, and negative regulators, such as Klotho and VDR [38]. Klotho, an antiaging protein, suppresses the expression of all RAS components both in vitro and in vivo [11], and vitamin D downregulates renin expression by inhibiting cyclic AMP-response element-binding protein [39]. Previously, we demonstrated that PM_2.5_ exposure during rat nephrogenesis caused glomerular and tubular injuries associated with increased oxidative damage and inflammation in the kidneys of dams and 3-week-old pups [25]. Pups from PM_2.5_-exposed dams exhibited significantly suppressed VDR, renin, and ACE expression in the kidneys at weaning [25]. In the current study, adult offspring from the maternal PM_2.5_ group showed reduced intrarenal VDR and Klotho expression but increased renin and ACE activities at 8 weeks old. The expression of vitamin D inactivating enzyme (CYP24A1) increased at weaning [25], whereas the activity of vitamin D activating enzyme (CYP27B1) was upregulated in adulthood. Maternal vitamin D supplementation restored VDR and ACE expression in the kidneys of 3-week-old pups [25] and reduced intrarenal ACE activity in 8-week-old adult offspring. Intrarenal ACE could be a key player in renal programming induced by maternal PM_2.5_ exposure, and vitamin D treatment may attenuate the aberrant activation of intrarenal RAS in adulthood caused by PM_2.5_ exposure during renal development. The loss of Klotho and VDR expression also appears to be associated with RAS activation in this context, although they were not restored by maternal vitamin D supplementation with PM_2.5_ exposure.

VDR plays a role in regulating genes involved in angiogenesis, inflammation, and fibrosis, as well as in suppressing the RAS [40,41]. Reduced VDR activation is observed from the very early stages of CKD in an animal model [40]. Exposure to a vitamin D-deficient diet during kidney development resulted in renal capillary rarefaction and persistently elevated renin activity, combined with upregulation of Ang-2 and downregulation of Ang-1/Tie-2 and VEGF/VEGFR2 in adulthood [21]. Vitamin D treatment attenuated renal damage by improving the Angs/Tie-2, VEGF/VEGFR2, and AT1 axes, as well as the transforming growth factor-β1/p-Smad2/3 signaling in a rat model of CKD [22]. Adult animals treated with vitamin D also showed improvements in renal structural and functional alterations caused by AT1 blockers during lactation [42]. Neonatal ACE inhibition upregulated intrarenal expression of VEGF-A, Ang-2, Tie-2, and TSP-1 while downregulating VEGFR1 and Ang-1 [43,44]. Glomerular and peritubular capillary endothelial cells stained with JG12 were substantially reduced in the ACE inhibitor-treated neonatal rat kidney [44]. In the present study, maternal exposure to PM_2.5_ during renal development induced elevated VEGF-A activity and reduced expressions of VEGFR2, Ang-1, and Tie-2, accompanied by increased glomerular and peritubular capillary loss in adult offspring rat kidneys. Vitamin D supplementation in dams improved capillary loss with reduced intrarenal VEGF-A levels and increased VEGFR2 and Ang-1 expression in adult offspring rats. In the previous [25] and current studies, we found that maternal PM_2.5_ exposure induced intrarenal RAS suppression in the neonatal stage and subsequent RAS activation along with microvascular rarefaction in adulthood. Both intrarenal RAS suppression in early life and its aberrant activation in adulthood can contribute to renal vascular homeostasis disruption and impair the angiogenic response throughout life.

In the kidney, the tubulovascular cross-talk between VEGF-A and VEGFR2 plays a key role in maintaining the peritubular microvasculature [10]. VEGF-A/VEGFR2 signaling during postnatal development is reported to be essential for the expansion of the renal medullary microcirculation [45]. Mice with insufficient VEGFR2 exhibited suppressed intrarenal VEGF-A/VEGFR2 signaling, peritubular capillary rarefaction, and kidney fibrosis [13]. Although VEGF-A expression shows a strong correlation with renal microvascular rarefaction and fibrosis during CKD progression, it remains unclear whether VEGF-A presence is beneficial or detrimental in renal fibrosis progression [7]. In the study of Kang et al. [46], VEGF-A expression was reduced in the medulla of the aging kidney, accompanied by peritubular capillary loss, yet it was focally increased in the cortex, seemingly as a compensatory response to impaired cortical perfusion. VEGF-A might be involved in both pro- and antifibrotic roles at different times and contexts in kidney diseases for the control of angiogenesis [7]. Furthermore, Klotho protein has been shown to inhibit the fibrotic response by reducing the expression of VEGF-A and transforming growth factor-β1/Smad3 [12]. Klotho deficiency causes a sustained elevation of VEGF-mediated intracellular [Ca^2+^] levels, leading to vascular hyperpermeability and extensive vascular calcification [14]. In Klotho-deficient mice, VEGFR2 deficiency exacerbated peritubular capillary loss, kidney dysfunction, and fibrosis [13]. In this study, upregulation of VEGF-A seems to be a compensatory response to the loss of glomerular and peritubular capillaries during repair and pathological processes. Loss of Klotho and VEGFR2 may contribute to capillary rarefaction and elevation of VEGF-A levels. Additionally, Ang-1/Tie-2 signaling, which is involved in stimulating angiogenesis and supporting vascular endothelial cell survival [47,48], was suppressed in adult rats exposed to maternal PM_2.5_. Reduced Ang-1/Tie-2 signaling may impair renal angiogenesis, despite elevated VEGF-A expression. Emerging evidence suggests that an increased ratio of Ang-2 to Ang-1, characterized by elevated Ang-2 and decreased Ang-1 levels, correlates with the pathophysiology of acute kidney injury, the transition from acute kidney injury to CKD, CKD, and cardiovascular events [47,48]. Collectively, this study demonstrates that early-life PM_2.5_ exposure resulted in long-term impairment of renal vascular homeostasis. In our experimental model, maternal PM_2.5_ exposure during kidney development induced aberrant activation of renin and ACE, depletion of VDR and Klotho, impairment of VEGF-A/VEGFR2 signaling, and inhibition of the Ang-1/Tie-2 axis in offspring rats later in life. Vitamin D treatment during pregnancy and lactation was able to reduce these vascular alterations in adult offspring rat kidneys.

The RAS regulation and vitamin D signaling pathway may play a crucial role in renal programming resulting from exposure to PM_2.5_ during kidney development. Our previous in vitro and in vivo studies suggest that prolonged renal effects from developmental PM_2.5_ exposure could stem from an imbalance in intrarenal RAS and vitamin D regulation [25,26]. Maternal vitamin D supplementation could alleviate the effects of renal programming induced by perinatal PM_2.5_ exposure. Yet, the safe dosage and optimal duration of vitamin D intake during pregnancy and lactation need further investigation, given that vitamin D can negatively regulate the RAS by inhibiting the renin gene, which is essential for fetal kidney development. Our study presents several limitations. The chemical composition and sources of PM_2.5_ change over time and space, potentially limiting the generalizability of our experimental model’s findings. While the sample size of three dams per group may be considered relatively small, this number was selected to minimize the sacrifice of female offspring. The exclusion of female offspring may also limit the translational relevance of the study, given the growing recognition of sex-specific differences in renal programming. Additionally, the glomerular counting method in our study does not fully reflect the total nephron number in the entire kidney, as it is based on partial counts from selected tissue sections. Thus, the findings should be interpreted with caution, considering potential variability such as differences in cortical region inclusion. Finally, the PM_2.5_ in this study was administered orally to the dams, creating a possibility that the concentration of PM_2.5_ in systemic circulation, absorbed through the gastrointestinal tract, might differ from that absorbed through the lungs. Nevertheless, PM_2.5_ inhaled into the lungs can cross the blood-gas barrier, subsequently affecting various organs and systems, including the kidneys [5,49]. Despite the limitations of our experimental models, the evidence suggests that early life exposure to PM_2.5_ during critical windows of susceptibility could cause persistent renal microvasculature alterations and nephron loss into adulthood. Maternal vitamin D treatment with PM_2.5_ exposure may reduce microvascular disturbance and preserve glomerular integrity through the regulation of intrarenal ACE, VEGF-A/VEGFR2, and Ang-1/Tie-2 axes.

## 5. Conclusions

Our results indicate that maternal exposure to PM_2.5_ during rat nephrogenesis leads to nephron and capillary loss, accompanied by the activation of renin and ACE, depletion of VDR and Klotho, impairment of VEGF-A/VEGFR2 signaling, and a reduction in Ang-1/Tie-2 expression in the kidneys of adult offspring. Maternal vitamin D supplementation restored VEGFR2 and Ang-1 activities as well as nephron and capillary architecture in adult offspring kidneys. Furthermore, maternal vitamin D intake, in the context of PM_2.5_ exposure, reduced intrarenal ACE and VEGF-A expressions. These findings provide novel insights into renal programming induced by early life PM_2.5_ exposure and suggest that maternal vitamin D supplementation may offer protective effects. Further research is needed to determine the optimal and safe dosage of vitamin D intake during pregnancy for offspring kidney health.

## Data Availability

The original contributions presented in this study are included in the article. Further inquiries can be directed to the corresponding author.

## Abbreviations

The following abbreviations are used in this manuscript:

Ang	angiopoietin
CKD	chronic kidney disease
CYP	cytochrome P 450 mixed-function oxidase
H&E	hematoxylin and eosin
PAS	periodic acid–Schiff
PM_2.5_	fine particulate matter
RAS	renin-angiotensin system
VEGF	vascular endothelial growth factor
VEGFR2	vascular endothelial growth factor receptor 2

## References

[1] Wathanavasin W., Banjongjit A., Phannajit J., Eiam-Ong S., Susantitaphong P. (2024). Association of fine particulate matter (PM_2.5_) exposure and chronic kidney disease outcomes: A systematic review and meta-analysis. Sci. Rep..

[2] Blum M.F., Surapaneni A., Stewart J.D., Liao D., Yanosky J.D., Whitsel E.A., Power M.C., Grams M.E. (2020). Particulate Matter and Albuminuria, Glomerular Filtration Rate, and Incident CKD. Clin. J. Am. Soc. Nephrol..

[3] Meariman J.K., Zulli H., Perez A., Bajracharya S.D., Mohandas R. (2023). Small vessel disease: Connections between the kidney and the heart. Am. Heart. J. Plus..

[4] Aztatzi-Aguilar O.G., Uribe-Ramírez M., Narváez-Morales J., De Vizcaya-Ruiz A., Barbier O. (2016). Early kidney damage induced by subchronic exposure to PM_2.5_ in rats. Part. Fibre Toxicol..

[5] Xu W., Wang S., Jiang L., Sun X., Wang N., Liu X., Yao X., Qiu T., Zhang C., Li J. (2022). The influence of PM2.5 exposure on kidney diseases. Hum. Exp. Toxicol..

[6] Xie G., Wang R., Yang W., Sun L., Xu M., Zhang B., Yang L., Shang L., Qi C., Chung M.C. (2022). Associations among prenatal PM_2.5_, birth weight, and renal function. Chemosphere.

[7] Miao C., Zhu X., Wei X., Long M., Jiang L., Li C., Jin D., Du Y. (2022). Pro- and anti-fibrotic effects of vascular endothelial growth factor in chronic kidney diseases. Ren. Fail..

[8] Min J. (2024). How to delay the progression of chronic kidney disease: Focusing on medications. Child. Kidney Dis..

[9] Abhinand C.S., Raju R., Soumya S.J., Arya P.S., Sudhakaran P.R. (2016). VEGF-A/VEGFR2 signaling network in endothelial cells relevant to angiogenesis. J. Cell Commun. Signal..

[10] Dimke H., Sparks M.A., Thomson B.R., Frische S., Coffman T.M., Quaggin S.E. (2015). Tubulovascular cross-talk by vascular endothelial growth factor a maintains peritubular microvasculature in kidney. J. Am. Soc. Nephrol..

[11] Zhou L., Mo H., Miao J., Zhou D., Tan R.J., Hou F.F., Liu Y. (2015). Klotho Ameliorates Kidney Injury and Fibrosis and Normalizes Blood Pressure by Targeting the Renin-Angiotensin System. Am. J. Pathol..

[12] Shin I.S., Shin H.K., Kim J.C., Lee M.Y. (2015). Role of Klotho, an antiaging protein, in pulmonary fibrosis. Arch. Toxicol..

[13] Shi M., Maique J.O., Cleaver O., Moe O.W., Hu M.C. (2023). VEGFR2 insufficiency enhances phosphotoxicity and undermines Klotho’s protection against peritubular capillary rarefaction and kidney fibrosis. Am. J. Physiol. Renal. Physiol..

[14] Kusaba T., Okigaki M., Matui A., Murakami M., Ishikawa K., Kimura T., Sonomura K., Adachi Y., Shibuya M., Shirayama T. (2010). Klotho is associated with VEGF receptor-2 and the transient receptor potential canonical-1 Ca^2+^ channel to maintain endothelial integrity. Proc. Natl. Acad. Sci. USA.

[15] Yildiz A.B., Copur S., Tanriover C., Yavuz F., Vehbi S., Gaipov A., Magagnoli L., Ciceri P., Cozzolino M., Kanbay M. (2024). Angiopoietin as a Novel Prognostic Marker in Kidney Disease. Blood. Purif..

[16] Potente M., Gerhardt H., Carmeliet P. (2011). Basic and therapeutic aspects of angiogenesis. Cell.

[17] Gnudi L., Benedetti S., Woolf A.S., Long D.A. (2015). Vascular growth factors play critical roles in kidney glomeruli. Clin. Sci..

[18] Madsen K., Marcussen N., Pedersen M., Kjaersgaard G., Facemire C., Coffman T.M., Jensen B.L. (2010). Angiotensin II promotes development of the renal microcirculation through AT1 receptors. J. Am. Soc. Nephrol..

[19] Feliers D., Gorin Y., Ghosh-Choudhury G., Abboud H.E., Kasinath B.S. (2006). Angiotensin II stimulation of VEGF mRNA translation requires production of reactive oxygen species. Am. J. Physiol. Renal. Physiol..

[20] Rizkalla B., Forbes J.M., Cooper M.E., Cao Z. (2003). Increased renal vascular endothelial growth factor and angiopoietins by angiotensin II infusion is mediated by both AT1 and AT2 receptors. J. Am. Soc. Nephrol..

[21] Ferreira de Almeida L., Della Coletta Francescato H., Antunes-Rodrigues J., Jose Albuquerque de Paula F., Giovanni Alves da Silva C., Silva Costa R., Machado Coimbra T. (2019). Imbalance of Pro- and Anti-Angiogenic Factors due to Maternal Vitamin D Deficiency Causes Renal Microvasculature Alterations Affecting the Adult Kidney Function. Nutrients.

[22] Deluque A.L., Oliveira B.M., Souza C.S., Maciel A.L.D., Francescato H.D.C., Giovanini C., de Almeida L.F., de Paula F.J.A., Costa R.S., Antunes-Rodrigues J. (2022). Paricalcitol Improves the Angiopoietin/Tie-2 and VEGF/VEGFR2 Signaling Pathways in Adriamycin-Induced Nephropathy. Nutrients.

[23] Hsu C.N., Tain Y.L. (2021). Targeting the Renin-Angiotensin-Aldosterone System to Prevent Hypertension and Kidney Disease of Developmental Origins. Int. J. Mol. Sci..

[24] Grigore D., Ojeda N.B., Robertson E.B., Dawson A.S., Huffman C.A., Bourassa E.A., Speth R.C., Brosnihan K.B., Alexander B.T. (2007). Placental insufficiency results in temporal alterations in the renin angiotensin system in male hypertensive growth restricted offspring. Am. J. Physiol. Regul. Integr. Comp. Physiol..

[25] Son M.H., Park E., Yim H.E., Nam Y.J., Lee Y.S., Choi E.K., Jeong S.H., Lee J.H. (2024). Maternal exposure to airborne particulate matter during pregnancy and lactation induces kidney injury in rat dams and their male offspring: The role of vitamin D in pregnancy and beyond. Kidney Res. Clin. Pract..

[26] Kang E., Yim H.E., Nam Y.J., Jeong S.H., Kim J.A., Lee J.H., Son M.H., Yoo K.H. (2022). Exposure to airborne particulate matter induces renal tubular cell injury in vitro: The role of vitamin D signaling and renin-angiotensin system. Heliyon.

[27] United States Environmental Protection Agency (2022). Exposure Assessment Tools by Routes—Inhalation [Internet]. https://www.epa.gov/expobox/exposure-assessment-tools-routes-inhalation#calculations.

[28] Ojeda N.B., Intapad S., Alexander B.T. (2014). Sex differences in the developmental programming of hypertension. Acta Physiol..

[29] Raij L., Azar S., Keane W. (1984). Mesangial immune injury, hypertension, and progressive glomerular damage in Dahl rats. Kidney Int..

[30] Ju L., Hua L., Xu H., Li C., Sun S., Zhang Q., Cao J., Ding R. (2023). Maternal atmospheric particulate matter exposure and risk of adverse pregnancy outcomes: A meta-analysis of cohort studies. Environ. Pollut..

[31] Morales-Rubio R.A., Alvarado-Cruz I., Manzano-León N., Andrade-Oliva M.D., Uribe-Ramirez M., Quintanilla-Vega B., Osornio-Vargas Á., De Vizcaya-Ruiz A. (2019). In utero exposure to ultrafine particles promotes placental stress-induced programming of renin-angiotensin system-related elements in the offspring results in altered blood pressure in adult mice. Part. Fibre Toxicol..

[32] Al-Odat I., Chen H., Chan Y.L., Amgad S., Wong M.G., Gill A., Pollock C., Saad S. (2014). The impact of maternal cigarette smoke exposure in a rodent model on renal development in the offspring. PLoS ONE.

[33] Popham K., Kandasamy Y. (2023). The impact of smoking and nicotine exposure during pregnancy on fetal nephrogenesis: A systematic review. J. Dev. Orig. Health Dis..

[34] Chien M.C., Huang C.Y., Wang J.H., Shih C.L., Wu P. (2024). Effects of vitamin D in pregnancy on maternal and offspring health-related outcomes: An umbrella review of systematic review and meta-analyses. Nutr. Diabetes.

[35] Woods L.L., Rasch R. (1998). Perinatal ANG II programs adult blood pressure, glomerular number, and renal function in rats. Am. J. Physiol..

[36] Machado F.G., Poppi E.P., Fanelli C., Malheiros D.M., Zatz R., Fujihara C.K. (2008). AT1 blockade during lactation as a model of chronic nephropathy: Mechanisms of renal injury. Am. J. Physiol. Renal. Physiol..

[37] Joung J., Cho H. (2023). Angiotensin receptor blocker induced fetopathy: Two case reports and literature review. Child. Kidney Dis..

[38] Yang T., Xu C. (2017). Physiology and Pathophysiology of the Intrarenal Renin-Angiotensin System: An Update. J. Am. Soc. Nephrol..

[39] Freundlich M., Quiroz Y., Zhang Z., Zhang Y., Bravo Y., Weisinger J.R., Li Y.C., Rodriguez-Iturbe B. (2008). Suppression of renin-angiotensin gene expression in the kidney by paricalcitol. Kidney Int..

[40] Xiong M., Gong J., Liu Y., Xiang R., Tan X. (2012). Loss of vitamin D receptor in chronic kidney disease: A potential mechanism linking inflammation to epithelial-to-mesenchymal transition. Am. J. Physiol. Renal. Physiol..

[41] de Borst M.H., Vervloet M.G., ter Wee P.M., Navis G. (2011). Cross talk between the renin-angiotensin-aldosterone system and vitamin D-FGF-23-klotho in chronic kidney disease. J. Am. Soc. Nephrol..

[42] de Almeida L.F., Francescato H.D.C., da Silva C.G.A., Costa R.S., Coimbra T.M. (2017). Calcitriol reduces kidney development disorders in rats provoked by losartan administration during lactation. Sci. Rep..

[43] Yim H.E., Kim J.H., Yoo K.H., Bae I.S., Hong Y.S., Lee J.W. (2011). Spironolactone and enalapril differentially up-regulate the expression of VEGF and heme oxygenase-1 in the neonatal rat kidney. Pediatr. Res..

[44] Yoo K.H., Yim H.E., Bae E.S., Hong Y.S. (2018). Capillary rarefaction and altered renal development: The imbalance between pro- and anti-angiogenic factors in response to angiotensin II inhibition in the developing rat kidney. J. Mol. Histol..

[45] Tinning A.R., Jensen B.L., Johnsen I., Chen D., Coffman T.M., Madsen K. (2016). Vascular endothelial growth factor signaling is necessary for expansion of medullary microvessels during postnatal kidney development. Am. J. Physiol. Renal. Physiol..

[46] Kang D.H., Anderson S., Kim Y.G., Mazzalli M., Suga S., Jefferson J.A., Gordon K.L., Oyama T.T., Hughes J., Hugo C. (2001). Impaired angiogenesis in the aging kidney: Vascular endothelial growth factor and thrombospondin-1 in renal disease. Am. J. Kidney Dis..

[47] Robinson-Cohen C., Katz R., Price B.L., Harju-Baker S., Mikacenic C., Himmelfarb J., Liles W.C., Wurfel M.M. (2016). Association of markers of endothelial dysregulation Ang1 and Ang2 with acute kidney injury in critically ill patients. Crit. Care.

[48] Li H., Song Q., Su X., Shen Y., Yan H., Yu Z., Li Z., Yuan J., Huang J., Ni Z. (2024). Serum angiopoietin-2/angiopoietin-1 ratio is associated with cardiovascular and all-cause mortality in peritoneal dialysis patients: A prospective cohort study. Ren. Fail..

[49] Zhang Y., Liu D., Liu Z. (2021). Fine Particulate Matter (PM2.5) and Chronic Kidney Disease. Rev. Environ. Contam. Toxicol..

